# A Situation Analysis of the Capacity of Laboratories in Faith-Based Hospitals in Zambia to Conduct Surveillance of Antimicrobial Resistance: Opportunities to Improve Diagnostic Stewardship

**DOI:** 10.3390/microorganisms12081697

**Published:** 2024-08-17

**Authors:** Doreen Mainza Shempela, Steward Mudenda, Maisa Kasanga, Victor Daka, Mundia Hendrix Kangongwe, Mapeesho Kamayani, Jay Sikalima, Baron Yankonde, Cynthia Banda Kasonde, Ruth Nakazwe, Andrew Mwandila, Fatim Cham, Michael Njuguna, Bertha Simwaka, Linden Morrison, Joseph Yamweka Chizimu, John Bwalya Muma, Roma Chilengi, Karen Sichinga

**Affiliations:** 1 Churches Health Association of Zambia, Lusaka 10101, Zambia; mapeesho.kamayani@chaz.org.zm (M.K.); jay.sikalima@chaz.org.zm (J.S.); baron.yankonde@chaz.org.zm (B.Y.); cynthia.kasonde@chaz.org.zm (C.B.K.); andrew.mwandila@chaz.org.zm (A.M.); karen.sichinga@chaz.org.zm (K.S.); 2 Department of Pharmacy, School of Health Sciences, University of Zambia, Lusaka 10101, Zambia; 3 Department of Pathology and Microbiology, University Teaching Hospitals, Lusaka 10101, Zambia; kasangaanita@gmail.com (M.K.); ruthnakazwe@yahoo.com (R.N.); 4 Department of Epidemiology and Biostatistics, School of Public Health, Zhengzhou University, Zhengzhou 450001, China; 5 Department of Public Health, School of Medicine, Copperbelt University, Ndola 10101, Zambia; dakavictorm@gmail.com; 6 Chest Diseases Laboratory, Ministry of Health, Lusaka 10101, Zambia; kangongwem@yahoo.com; 7 Global Fund to Fight AIDS, Tuberculosis and Malaria (GFATM), 1201 Geneva, Switzerland; fatim.jallow@theglobalfund.org (F.C.); michael.njuguna@theglobalfund.org (M.N.); Bertha.Simwaka@theglobalfund.org (B.S.); linden.morrison@theglobalfund.org (L.M.); 8 Antimicrobial Resistance Coordinating Committee, Zambia National Public Health Institute, Lusaka 10101, Zambia; chizimuyjoseph@yahoo.com (J.Y.C.); chilengir@yahoo.com (R.C.); 9 Department of Disease Control, School of Veterinary Medicine, University of Zambia, Lusaka 10101, Zambia; jmuma@unza.zm

**Keywords:** antimicrobial resistance, bacteriology, laboratory capacity, surveillance, Zambia

## Abstract

Antimicrobial resistance (AMR) is a public health problem exacerbated by the overuse and misuse of antibiotics and the inadequate capacity of laboratories to conduct AMR surveillance. This study assessed the capacity of laboratories in seven faith-based hospitals to conduct AMR testing and surveillance in Zambia. This multi-facility, cross-sectional exploratory study was conducted from February 2024 to April 2024. We collected and analysed data using the self-scoring Laboratory Assessment of Antibiotic Resistance Testing Capacity (LAARC) tool. This study found an average score of 39%, indicating a low capacity of laboratories to conduct AMR surveillance. The highest capacity score was 47%, while the lowest was 25%. Only one hospital had a full capacity (100%) to utilise a laboratory information system (LIS). Three hospitals had a satisfactory capacity to perform data management with scores of 83%, 85%, and 95%. Only one hospital had a full capacity (100%) to process specimens, and only one hospital had good safety requirements for a microbiology laboratory, with a score of 89%. This study demonstrates that all the assessed hospitals had a low capacity to conduct AMR surveillance, which could affect diagnostic stewardship. Therefore, there is an urgent need to strengthen the microbiology capacity of laboratories to enhance AMR surveillance in Zambia.

## 1. Introduction

Antimicrobial resistance (AMR) occurs when bacteria, viruses, fungi, and parasites do not respond to antimicrobial drugs in animals or humans, hence allowing the organisms to survive in the host despite exposure to sufficient drug doses [1,2,3]. Antimicrobial resistance (AMR) has been spreading extensively worldwide and remains a serious threat to public health globally [4,5,6]. The effects of AMR cut across health, environmental, and economic paradigms, making its impact more pronounced [7,8,9]. This phenomenon has been worsened by a lack of surveillance and diagnostic systems, especially in low-income settings, where large quantities of antimicrobial agents are used [10,11,12]. With the problem expected to escalate, it is estimated that there will be 10 million deaths per year globally due to AMR [1,13].

The deficiency in laboratory capacity and systems to conduct AMR surveillance has been linked to many factors, including a lack of funding, inadequate training of healthcare personnel, a lack of training in antimicrobial stewardship (AMS), shortages of laboratory consumables, and a lack of necessary laboratory equipment [14]. Consequently, some laboratories in low-resource settings face challenges regarding infrastructure, technical issues, and behavioural change in implementing clinical bacteriology testing [15]. Additionally, evidence has shown that the role of laboratories in the surveillance of AMR is affected by the absence of antibiograms, a lack of quality management systems, a lack of proficiency testing, and a lack of guidelines and standard operating procedures (SOPs), among other challenges [7,15,16,17,18]. A study conducted at a teaching hospital in Ghana highlighted the importance of cumulative antibiograms in AMS programs and their influence on prescribing practices among clinicians [19].

Strengthening the laboratory surveillance of AMR is essential to address escalating drug-resistant infections [20,21,22,23]. Studies have shown an improvement in AMR surveillance after improving the capacity of laboratory testing [7,11,24]. Additionally, laboratory findings help clinicians to make decisions regarding the treatment of particular infections [14,25,26]. Therefore, this promotes the principles of diagnostic stewardship through which laboratories contribute to the appropriate diagnosis and treatment of infectious diseases [27,28,29,30]. Hence, through diagnostic stewardship, antimicrobials are prescribed based on the correct test to the correct patient, which prompts the correct action, leading to the efficient use of resources, the improved use of antimicrobials, and a reduction in AMR [27,28,31,32]. However, there are some concerns about clinicians underutilising clinical microbiology laboratory data when making clinical decisions [33].

Substantial investments have to be made to improve laboratory capacity to conduct AMR surveillance, especially in low-income and middle-income countries (LMICs), which have the highest burden of diseases [34,35]. Evidence has shown that the surveillance of AMR in LMICs, especially in African countries, is faced with many challenges that have an impact on disease diagnosis and treatment [10,36,37]. These challenges also lead to poor clinical care and patient outcomes [38]. The low capacity of laboratories to conduct surveillance of AMR has been reported in some African countries [10,39,40,41]. Additionally, the surveillance of AMR in African countries is affected by inadequate data collection, analysis, reporting, and sharing; poor collaborations among stakeholders; and a lack of laboratory capacity to conduct AMR surveillance [42]. Therefore, there is a need for African countries to mobilise resources and strengthen collaborations with partners and stakeholders to address AMR [42].

Addressing AMR requires the systematic initiation and implementation of AMS programs in healthcare facilities [43,44,45]. Through AMS programs, laboratory staff can be educated and trained in good laboratory practices that aim to promote the generation of high-quality and reliable test data, a reduction in antimicrobial use, and a reduction in AMR [19,46,47,48]. AMS programs can facilitate education and capacity building among laboratory scientists, leading to enhanced antimicrobial susceptibility testing (AST), enhanced culture and susceptibility reports, accurate rapid diagnostic testing, and improved alert and surveillance systems [49]. Furthermore, collaborations that foster action-oriented and multidisciplinary-facility AMS programs can lead to the successful implementation of AMS activities [50,51]. Notably, AMS interventions lead to improved prescribing practices of antimicrobials and lead to improved patient outcomes [52,53,54,55,56].

Zambia is a country in the sub-Saharan African (SSA) region with a reportedly high burden of infectious diseases [57,58,59]. Additionally, the presence of drug-resistant pathogens in healthcare facilities has been reported in several studies [60,61,62,63,64,65,66,67,68]. The Antimicrobial Resistance Coordinating Committee (AMRCC) hosted at the Zambia National Public Health Institute (ZNPHI) has been promoting the establishment and strengthening of AMR surveillance across the country [69,70]. The ZNPHI has supported One Health AMR surveillance through the 2019 Zambia Integrated Surveillance Framework for Antimicrobial Resistance [69]. However, there is a paucity of information on the capacity of laboratories to conduct AMR surveillance in Zambia. Therefore, this study assessed the capacity of seven faith-based hospitals to conduct bacteriology, AMR testing, and surveillance in Zambia. It was envisaged that the findings of this assessment would be used to strengthen AMR surveillance in faith-based hospital-affiliated laboratories in Zambia.

## 2. Materials and Methods

### 2.1. Study Design, Period, and Setting

**Figure 1:**
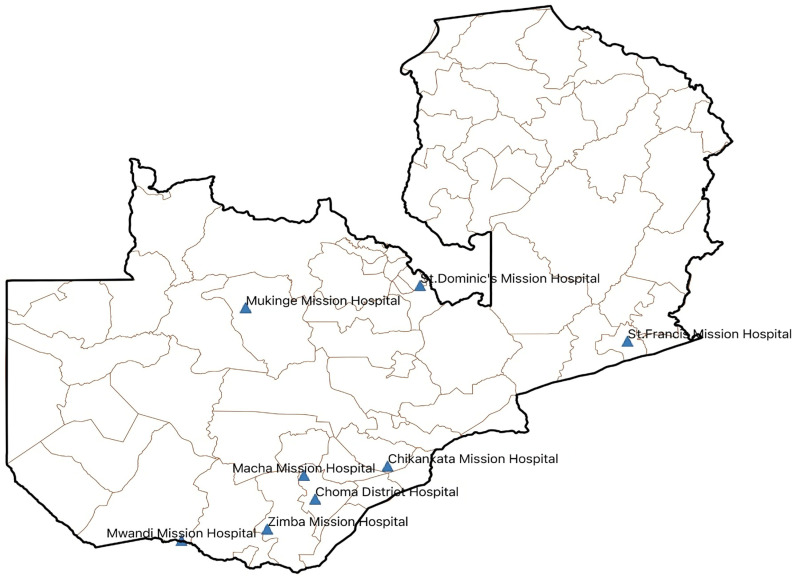
Map of Zambia showing the location of faith-based health facilities included in the baseline assessment study.

We conducted a cross-sectional exploratory study in seven faith-based hospitals in Zambia from February 2024 to April 2024. We used an exploratory research approach because there was a lack of information on this topic due to few or no studies conducted in Zambia on the subject matter. The hospitals are located in five different provinces, as follows: Chikankata Mission Hospital (CMH) in the Chikankata District, Macha Mission Hospital (MaMH) in the Choma District, Zimba Mission Hospital (ZMH) in the Zimba District of the Southern Province, Mwandi Mission Hospital (MMH) in the Mwandi District of the Western Province, St. Francis Mission Hospital (SFMH) in the Katete District of the Eastern Province; St. Dominic’s Mission Hospital (SDMH) in the Ndola District of the Copperbelt Province; and Mukinge Mission Hospital (MuMH) in the Kasempa District of the North-Western Province. These faith-based hospitals were chosen because they provide medical and surgical services to many Zambian people, largely in remote places (Figure 1). Additionally, the sites were selected because they are under the ambit of the Churches Health Association of Zambia (CHAZ), which will strengthen the microbiology activities of these hospitals to help scale up their capacity to conduct bacteriology, AMR testing, and surveillance. In our study, a faith-based hospital was defined as a hospital supported by or affiliated with a Christian religious group such as Catholics, Anglicans, Presbyterians, or Protestants.

### 2.2. Sample Size and Sampling Technique

We selected seven faith-based hospitals using the purposive sampling method to conduct a baseline assessment of the capacity of laboratories to perform basic medical bacteriology, AMR testing, and surveillance. Purposive sampling has been used in studies similar to the current one because it helps to collect comprehensive, specific information based on the study objectives [71,72]. We purposefully selected four key informants working in the microbiology section of the laboratory in the surveyed hospitals. Purposive sampling was used because it helps to select participants who have a specific characteristic to be included in a study. This provided us with a total of 28 key informants across the seven hospitals. The key informants included the laboratory manager, the quality control officer, and bench personnel.

### 2.3. Data Collection

Data collection for this study was conducted using a Microsoft Excel sheet version of the self-scoring Laboratory Assessment of Antibiotic Resistance Testing Capacity (LAARC) questionnaire [73]. The tool was validated and recommended by the Centres for Disease Control and Prevention (CDC) for evaluating laboratories proficient in conducting bacteriologic techniques and other related quality processes that require accurate and reliable AMR detection and reporting [73]. In this study, we adopted this tool because of its technical depth and granularity, validation by the CDC, and design intended to be used in limited-resource settings [73]. The LAARC questionnaire has 15 modules, each containing 3 to 10 indicators. Additionally, each indicator contains closed-ended questions to which laboratory personnel respond [73].

The 15 modules include Module 0, called general (laboratory demographics, test menu and workload, ST/AMR workload and methods, laboratory staff education and training, QMS mentoring programs, and accreditation and certification); Module 1: facility (laboratory facility, general equipment availability, media preparation equipment availability, equipment calibration records, thermometers, temperature and atmosphere monitoring, autoclave management, instrument availability and maintenance, and inventory and stock outs); Module 2: laboratory information system (LIS) (demographic data fields, specimen data fields, culture observation data fields, AST data fields, reports and data transfer capabilities, and interface connectivity); Module 3: data management (patient and specimen identification, specimen requisition form, order entry, culture observations, AST result reporting, data backup and security, and AMR data sharing); Module 4: quality assurance (QA) (quality structure/basics, laboratory staff education/training/competency, troubleshooting, problem solving, route cause analyses, and external quality assessment (EQA); Module 5: media quality control (QC Media) (media preparation SOPs, general media preparation, distilled/deionised water preparation, routine media QC, Mueller–Hinton media preparation and QC, and blood culture bottle preparation and QC); Module 6: identification QC (QC ID) (gram stain QC and reagent labelling and storage, QC of individual biochemical methods, QC of enteric serology, and QC of commercial ID kids and automated ID systems); Module 7: antimicrobial sensitivity testing QC (QC AST) (routine AST reference strains, special AST reference strains, QC of disc diffusion methods, QC of gradient strip AST methods, and QC of automated AST systems); Module 8: specimen (specimen management, specimen rejection, blood specimen collection and transportation, urine collection specimen and transportation, and stool specimen collection and transportation); Module 9: processing (blood culture processing, manual blood culture systems, urine culture, stool cultures for Salmonella and Shigella); Module 10: identification methods and standard operating procedures; Module 11: basic AST (antibiotic disc and gradient strip maintenance, inoculum preparation, inoculum/incubation, reading AST results, interpreting AST results, and breakpoint standards; Module 12: AST expert rules (expert rules for Salmonella, Gram-negative, and beta-lactam breakpoints; phenotypic ESBL testing; phenotypic carbapenamase testing; colistin testing; expert rules for *Staphylococcus aureus*; general considerations and expert rules for Streptococcus pneumoniae; inducible clindamycin resistance testing; and expert rules for cerebrospinal fluid); Module 13: AST policy (AST panels, cumulative antibiograms, and AST policy); and Module 14: safety (biosafety equipment, personal protective equipment, biosafety behaviours, and biosafety documentation and training) [73].

The data collection team made a courtesy call to the hospital management and explained the purpose of the assessment. The support that the data collecting team received from the hospital management in the surveyed hospitals made data collection easier and without challenges. After that, the team visited the laboratory and interacted with laboratory staff regarding the purpose of their visit. The team toured the laboratory and reviewed documents, including SOPs. The questionnaire was administered for two days (approximately eight hours per day) per facility by four data collectors, including two public health experts and two clinical microbiology experts. A summary of the findings was to be prepared and shared with the hospital management and laboratory staff. Since the facilities are sparsely distributed, the data were collected for 18 days.

### 2.4. Data Analysis

The LAARC tool is a self-scoring tool; therefore, the data were entered into the LAARC tool for initial scoring based on each indicator. The minimum score was 0% and the maximum score was 100%. Low capacity to conduct basic medical bacteriology and test for AMR scored from 0 to 49%, moderate capacity was scored 50–79%, and good capacity was scored 80–100% [73]. All the charts were developed using Microsoft Excel version 2013. The overall scores for the hospitals were generated, and an average was calculated to produce the overall capacity of the laboratory to conduct AMR surveillance. For the assessed indicators, the scores were aggregated for all hospitals, and an average score was generated to indicate the capacity of laboratories to perform activities under that indicator. Scores of 0–49% meant that the laboratory needed significant improvement in conducting the activities under such indicators. Scores of 50–79% denoted that improvements were required, while scores of 80% and above were satisfactory (good capacity).

## 3. Results

**Figure 2:**
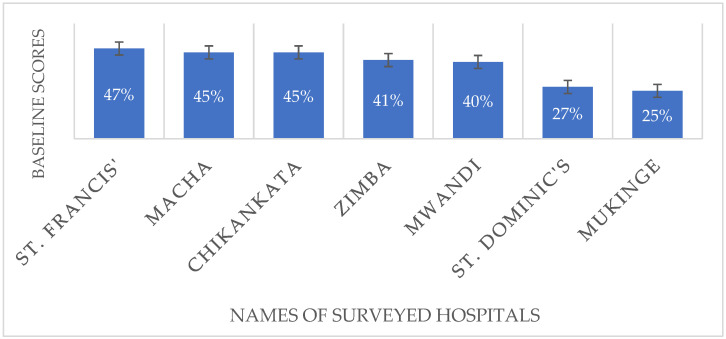
Overall capacity to conduct bacteriology, testing for antimicrobial resistance, and surveillance among faith-based hospitals in Zambia.

This study enrolled seven faith-based hospitals in five provinces of Zambia. All seven hospitals surveyed had physical laboratory infrastructure. However, they all scored below 50% regarding their ability to conduct basic medical bacteriology and capacity to test for AMR, indicating low capacity to conduct AMR surveillance (Figure 2). The average score of laboratories to conduct AMR surveillance was 39%, with St. Francis Mission Hospital having the highest overall score (Figure 2).

**Figure 3:**
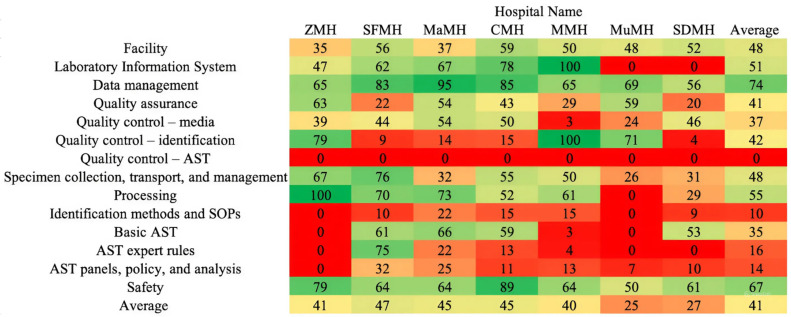
Baseline scores of hospitals regarding their capacity to conduct basic medical bacteriology and AMR testing based on the assessed indicators. Note: ZMH = Zimba Mission Hospital, SFMH = St. Francis Mission Hospital, MMH = Macha Mission Hospital, CMH = Chikankata Mission Hospital, MMH = Mwandi Mission Hospital, MuMH = Mukinge Mission Hospital, and SDMH = St. Dominic’s Mission Hospital.

This study found that an average score of 41% was obtained for all the assessed indicators, thereby demonstrating the low capacity of hospitals to conduct medical bacteriology and AMR testing (Figure 3). The surveyed hospitals scored low (0–48%) in 10 indicators and moderate (51–74%) in 4 indicators (Figure 3). The highest overall score was 74% regarding data management, with only St. Francis Mission Hospital (SFMH), Macha Mission Hospital (MaMH), and Chikankata Mission Hospital (CMH) having performed well in this indicator. All the surveyed hospitals scored 0% in quality control of ASTs (Figure 3). Only CMH performed well regarding the safety audit. Finally, only Mwandi Mission Hospital (MMH) had good capacity to run LIS and to conduct identification quality control (Figure 3). Individual facility scores are also shown in the Supplementary Material (Excel S1).

## 4. Discussion

This study was a situation analysis involving a baseline assessment of the capacity of laboratories to conduct medical bacteriology, AMR testing, and surveillance in faith-based hospitals in Zambia. All seven faith-based hospitals exhibited limited capacity to conduct AMR surveillance, with an average score of 39%. Additionally, an average score of 41% was recorded based on the assessed indicators. Despite having physical laboratory infrastructure across all seven hospitals, only three had good capacity to perform data management, only one hospital had a well-functioning LIS, and only one hospital met the safety standards required of a microbiology laboratory. These findings may negatively affect diagnostic stewardship and patient outcomes. Therefore, our findings demonstrated a strong need to strengthen the capacity of laboratories to perform bacteriology, AMR testing, and surveillance.

Our study found a low capacity of faith-based hospital-affiliated laboratories to conduct medical bacteriology, AMR testing, and the implementation of laboratory-based AMR surveillance in Zambia. Our findings are evidenced by the low average score regarding the indicators that we used in assessing the capacity of laboratories to test for AMR and perform all bacteriological processes. Therefore, these findings indicate a weakness of the surveyed laboratories in conducting AMR surveillance, which could affect disease diagnosis and treatment. These findings align with those reported in other studies [7,40,41,71]. Another study in Burkina Faso found that the overall capacity to conduct AMR surveillance was 40% across 18 laboratories, with the highest laboratory scoring 58% and the lowest 26%, indicating a low capacity to conduct QMS and AMR surveillance [39]. The low capacity of laboratories to conduct bacteriological tests and AMR testing affects the surveillance of AMR. This problem affects many other African countries [42]. In Africa, the lack of capacity of laboratories to conduct most microbiology tests affects the validity, usefulness, and trustworthiness of data generated from the surveillance systems [37]. The low capacity of laboratories to conduct AMR surveillance is an opportunity for health authorities and partners to strengthen and improve diagnostic stewardship and address AMR [74].

In this study, none of the surveyed laboratories had a full capacity to test for AMR due to challenges including sub-optimum laboratory conditions, equipment unavailability, a lack of calibration and maintenance, inconsistent or unavailable temperature monitoring, a lack of an autoclave, and inconsistent or absent inventory management. Our findings resonate with another study conducted in Ethiopia where, despite the availability of laboratories, there was a lack of basic equipment and consumables to conduct AMR testing [7]. Another study in Kenya reported similar findings, where nearly one-third of the healthcare facilities examined did not offer bacterial culture testing, and only a meagre 16.9% conducted AST [41]. This was due to poor access to LISs, low participation in external quality assessment programs for cultures, severe infrastructural gaps, and a lack of equipment [41]. A study in Rwanda also reported that critical gaps in equipment and supplies were found in district hospitals [75]. Our findings and those reported in other African countries conform with earlier findings that there is a lack of proper, reliable, cost-effective, and easy-to-use AMR diagnostic tools, particularly in routine diagnostic laboratories [38,42]. Additionally, there are few reports of major testing gaps for AMR in other regions with currently available data comprehensively describing AMR trends [76]. These differences could be attributed to differences in the setup and development of healthcare systems, particularly those in the African region severely impacted by financial constraints and infrastructural challenges.

Our study revealed that only Mwandi Mission Hospital had full LIS capacity to run electronic data management systems, while the St. Francis, Macha, and Chikankata Mission Hospitals had moderate capacity. This finding indicates a strength that Mwandi Mission Hospital possessed regarding LISs. The Mukinge and St. Dominic’s Mission Hospitals could not run LIS, demonstrating a critical disadvantage in the processing and storage of information. Our findings corroborate those reported in Kenya, where most laboratories scored low on the LIS component of the indicators [41]. Evidence has shown that LIS access is generally poor in most LMICs, affecting AMR surveillance [34]. Studies have reported that laboratories need to develop and implement their LISs to reduce identification errors, improve data security and protection, and promote the efficiency of reporting results [77,78,79].

The current study found that only three hospitals, including the Chikankata, St. Francis, and Macha Mission Hospitals, had good data management capacity and scored well in patient and specimen identification, specimen requisition forms, order entry, culture observations, AST data reporting, data backup and security, and AMR data sharing. Hence, four hospitals in this study had weaknesses, as they could not perform effective data management. These findings were also echoed in a study in Ethiopia, where data capture and transmission challenges were cited as common problems in implementing AMR surveillance work [80]. Additionally, a comprehensive review underscored the necessity for prompt intervention to address the issues related to data infrastructure and financial stability [81]. This is crucial to maintain the effectiveness and sustainability of surveillance initiatives. It has been established that data systems for AMR surveillance are essential for fostering unified public health measures and guaranteeing the distribution of sufficient feedback [81]. Our findings agree with those of a previous study conducted in Nepal, where data generated from facilities were inconsistent, incomplete, and delayed, hence causing challenges with data transmitted to the national level for action [24]. In contrast, a study in Ethiopia described effective data management after deliberate efforts to establish a surveillance system [82]. These differences could be due to an absence of systems for capturing AMR data. Additionally, a lack of training on data management and AMR could be one of the barriers that affected effective data management in some hospitals.

In our study, all the surveyed hospitals could not perform quality assurance, including the basics of the quality management system (QMS), staff competency assessments, troubleshooting mechanisms, and external QA. The implementation of QMS dictates that health facilities align themselves to national and international requirements of quality assurance, which eventually enable a culture of quality in all sectors of healthcare [18]. A study among 18 laboratories in Burkina Faso also reported a low performance of laboratories to conduct QMS and external QA [39]. Not surprisingly, our study found that all the surveyed hospitals could not perform media QC, identification QC, or AST QC. Similarly, a study in Togo had findings in which the AST QC was very low [83]. In contrast to our findings, a study in Burkina Faso found that most (76.5%) facilities had a good capacity to conduct AST QC [39]. In keeping with the findings from the Burkina Faso study [39], a study in Kenya reported good performance (≥80%) in QA, and a score of 81.1% in AST QC was reported [41]. Intriguingly, evidence has shown that implementing QMS in laboratories enhances patient safety [84].

All the surveyed hospitals in the current study did not have the full capacity to implement bacteriological identification methods. The methods used were not supported by SOPs, protocols, or flow charts. These findings are worrying because a lack of the proper identification of microorganisms affects the management, treatment, and control of infections [85]. A lack of SOPs, protocols, and flow charts has also been reported in other countries, especially in low-resource settings [15,17,18]. Implementing clinical bacteriology improves patient management, provides valuable surveillance for local antibiotic treatment guidelines, and supports the containment of AMR and the prevention and control of hospital-acquired infections [15]. SOPs contain instructions on how to isolate and identify pathogens, assign bacteria to a specific group, interpret results, and recommend panels of antibiotics for AST for a particular pathogen [17,86]. Hence, gaps in basic identification methods and a lack of SOPs negatively affect the process of bacteriology. There is an urgent need to strengthen the laboratory detection of pathogens by improving the laboratory capacity to conduct bacteriology [85]. Therefore, the need for SOPs in bacteriological processes must be emphasised in maintaining the accuracy, consistency, and quality of data [86].

Our study revealed that none of the seven hospitals had the full capacity to perform the maintenance of discs and strips, inoculum preparation, incubation, and reading and interpreting results and breakpoint standards. Hence, this deficiency and weakness highlight the potential problems associated with adhering to the established guidelines provided by the Clinical and Laboratory Standards Institute (CLSI), the European Committee on Antimicrobial Susceptibility Testing (EUCAST) breakpoints and the AST expert rules for the priority pathogens. These challenges are mostly seen in LMICs, where securing the supply chain and investing in adapted equipment, diagnostics, and reagents adapted to the environmental, logistic, and financial challenges is difficult to sustain [87]. In addition, none of the seven hospitals in the present study had the full capacity to perform specimen collection, transport, and management, or the processing of blood, urine, and stool cultures. Many studies have documented challenges in conducting routine bacteriological diagnostics and subsequent AST, mainly in low- and middle-income countries [14,76,80,82,88].

In this study, only Chikankata Mission Hospital had a good capacity for the safety requirements of a microbiology laboratory that must be involved in AMR testing and surveillance. Zimba Mission Hospital was at borderline (79%) to attain good capacity regarding safety audits in the microbiology laboratory. Poor safety standards in laboratories put laboratory workers at an increased risk of contracting laboratory-acquired infections [89]. The challenges in biosafety and biosecurity have been documented in other countries, outlining the lack of safety assurance leading to the unsafe handling of infectious materials in the laboratory [90,91]. Ultimately, this has affected the implementation of AMR programs in health facilities and laboratories [92,93]. Our findings and those reported in other studies indicate the need to conduct frequent assessments and monitoring of biosafety and biosecurity in laboratories [94]. Hence, there is a critical need to strengthen biorisk management in laboratories to reduce and tackle infections [89,95].

We found that none of the seven assessed laboratories had an antibiogram. The absence of antibiograms is a weakness which creates a huge gap in promoting targeted antimicrobial treatment and affects the feasibility of evaluating the extent of pathogen resistance to antimicrobials. Notably, using antibiograms enhances the rational use of antimicrobials and promotes AMS [19,96,97]. Additionally, antibiograms are used to detect and monitor current patterns in AMR and decrease the overall use of antibiotics empirically [98,99]. The lack of antibiograms in clinical laboratories can have substantial implications for patient treatment and public health. The significance of antibiograms in the presence of significant AMR in Gram-negative bacteria has been underscored, focusing on their role in directing antibiotic treatment and managing multi-drug-resistant organisms [100]. Notably, antibiograms are essential in enhancing AMS, infection control, and rational antimicrobial therapy [19,101]. A study in Ghana highlighted the importance of antibiograms’ ability to support empirical clinical decision-making and enhance infection prevention, highlighting their value in laboratories [102].

The gaps identified in our study demonstrate the need to develop strategies that should be used to improve the surveillance of AMR in Zambia. In addition, none of the surveyed hospitals had an AMS committee to champion heightened AMR surveillance. Notably, this is an opportunity for the AMS implementers in Zambia to establish stewardship programs in faith-based hospitals. Therefore, we believe setting up AMS teams is critical to improving the surveillance of AMR, similar to earlier publications [21,103,104,105,106,107,108,109]. Additionally, AMS and surveillance programs can help in capacity building and the mentorship of laboratory staff regarding microbiology and AMR surveillance [49]. We also believe that the surveyed hospitals could benefit from partnerships with other organisations and institutions involved in the fight against AMR and implementing AMS programs. Previous studies have reported the importance of strengthening the fight against AMR through partnerships [110,111,112,113,114]. Hence, strengthening the laboratory capacity to monitor and conduct AMR surveillance potentially reduces the development and spread of AMR [74,115,116,117]. It is thus imperative to consider many areas when strengthening laboratory capacity to conduct AMR surveillance, including laboratory location, ownership, access to clinical and prescription databases linked to the laboratory, improved LIS or digitised AMR data, the collaborative networks and spirit of a laboratory, laboratory working environment and equipment, laboratory staffing, quality assurance and assessments, laboratory methods and specimen types, data completeness and cleanliness, and the quantity of AMR data [114].

We are aware that our study had limitations. First, this was a cross-sectional study relying on self-reporting by respondents and is prone to recall biases. Second, this study was conducted in faith-based hospitals. Hence, the generalisation of the findings must be performed cautiously, as the findings may not reflect what is obtained in public and private hospitals. Therefore, the study findings may not represent all the faith-based hospitals in Zambia, as the study was performed in five out of ten provinces in Zambia. However, our findings are vital and can be used to identify opportunities and gaps that can be leveraged to strengthen AMR surveillance in faith-based hospitals in Zambia. As a starting point to improve AMR testing and surveillance in the surveyed hospitals, the presence of laboratory infrastructure in all seven hospitals is very cardinal. We provided recommendations to all the hospitals for strengthening the laboratory surveillance of AMR by addressing all the gaps identified in this baseline study. Finally, we will conduct follow-up studies to improve the capacity of laboratories to conduct AMR surveillance, improve diagnostic stewardship, and assess the impacts of interventions. Before follow-up studies, mentorship and educational programs including microbiological techniques; pathogen identification; conducting biochemical tests and ASTs; using SOPs and other guidelines; specimen collection, transportation, storage, and processing; laboratory safety rules; awareness of AMR; surveillance; AMS; and diagnostic stewardship will be conducted in the selected hospitals to help equip the laboratory staff with knowledge and skills related to AMR surveillance.

## 5. Conclusions

This study demonstrates that the assessed hospitals had a low capacity regarding the testing and surveillance of AMR. The presence of a physical laboratory in all seven hospitals is important for setting up and implementing AMR testing and surveillance. There is a need to strengthen all seven laboratories in areas of laboratory conditions, equipment, LISs, data management, quality control, specimen collection and handling, pathogen identification methods, the conducting of AST, and safety standards. These improvements could in turn improve diagnostic stewardship and patient outcomes. Further, this strategy can be extended to other laboratories across the country. Furthermore, we will conduct follow-up studies to assess improvements in bacteriological testing and AMR surveillance in the hospitals where baseline studies were conducted.

## Data Availability

The data supporting the reported results can be made available on request from the corresponding author.

## Supplementary Materials

The following supporting information can be downloaded at: https://www.mdpi.com/article/10.3390/microorganisms12081697/s1, Microsoft Excel Document S1.
